# Supplementation with Bromelain, Troxerutin, and Escin to Support Postoperative Recovery After Hip or Knee Arthroplasty in Older Adults: A Pilot Study

**DOI:** 10.3390/nu17243815

**Published:** 2025-12-05

**Authors:** Francesco Landi, Matteo Tosato, Roberta Terranova, Giulia Rubini, Federica Mammarella, Stefano Cacciatore, Emanuele Marzetti, Anna Picca, Hélio José Coelho-Júnior, Riccardo Calvani

**Affiliations:** 1 Department of Geriatrics, Orthopedics and Rheumatology, Università Cattolica del Sacro Cuore, Largo Francesco Vito 1, 00168 Rome, Italy; roberta.terranova02@icatt.it (R.T.); giulia.rubini01@icatt.it (G.R.); coelhojunior@hotmail.com.br (H.J.C.-J.); riccardo.calvani@unicatt.it (R.C.); 2 Fondazione Policlinico Universitario “Agostino Gemelli” IRCCS, Largo Agostino Gemelli 8, 00168 Rome, Italy; matteo.tosato@policlinicogemelli.it (M.T.); picca@lum.it (A.P.); 3 Department of Medicine and Surgery, LUM University, Strada Statale 100 Km 18, 70010 Casamassima, Italy

**Keywords:** bromelain, postoperative pain, postoperative edema, orthopedic rehabilitation, older adults

## Abstract

**Background:** Bromelain, a proteolytic enzyme extracted from *Ananas comosus*, exhibits anti-edematous and anti-inflammatory properties that may facilitate postoperative recovery. Troxerutin and escin, respectively, a vasoactive flavonoid and a saponin derivative, also provide anti-edematous and microcirculatory benefits that could enhance tissue repair and functional outcomes. Evidence on their combined use in older adults undergoing rehabilitation after major orthopedic surgery remains limited. **Methods**: We conducted retrospective observational study in adults aged 65 years or older admitted to a post-acute rehabilitation unit after total hip or total knee arthroplasty. Half of the participants received an oral supplement containing bromelain (400 mg/day), troxerutin (300 mg/day), and escin (40 mg/day) for up to 21 days alongside usual care and standard medications. The primary outcome was pain reduction assessed through the Visual Analog Scale (VAS). Secondary outcomes included changes in postoperative edema and functional recovery, evaluated through range of motion, the Barthel Index, and gait performance. **Result:** Forty participants were enrolled (mean age 69.4 ± 7.2 years; 58 percent women). Individuals receiving the combined supplement achieved significantly greater pain improvement than controls. At day 10 (T1), VAS scores declined from 6.8 ± 1.0 to 3.2 ± 0.9 in the supplemented group versus 6.7 ± 1.1 to 4.5 ± 1.0 in controls (*p* < 0.01). At day 21 (T2), VAS further decreased to 1.8 ± 0.7 in the supplemented group and to 3.1 ± 0.8 with standard treatment (*p* < 0.001). Functional performance also improved more markedly with supplementation, with earlier mobilization and faster recovery of autonomy. No significant side effects were reported. **Conclusions:** In this pilot study, combined bromelain, troxerutin, and escin supplementation was associated with meaningful reductions in postoperative pain and edema and with faster functional recovery. Larger controlled trials are warranted to confirm these effects and elucidate underlying mechanisms.

## 1. Introduction

Total hip and knee arthroplasty are among the most effective and widely performed orthopedic procedures worldwide, designed to improve mobility, reduce pain, and enhance the overall quality of life in individuals with progressive degenerative joint diseases. However, even after successful surgery, the immediate postoperative phase is often characterized by pain, inflammatory swelling, and temporary functional limitations that can influence the progress of rehabilitation. Despite substantial technical advances in surgical procedures and developments in prosthetic design in recent years, the postoperative phase remains a critical period in which pain, inflammation, and functional impairment may negatively affect rehabilitation outcomes [1,2]. During the postoperative period, the inflammatory response to surgical procedures plays an essential role in tissue repair but may also become disproportionate, leading to excessive edema, uncontrolled pain, and functional limitations that delay recovery and prolong hospital stay [3]. For this reason, adequate control of post-surgical inflammation, typically achieved through the use of nonsteroidal anti-inflammatory drugs (NSAIDs), represents a fundamental component of care and must be integrated within rehabilitation programs. However, the prolonged use of NSAIDs and other analgesic agents is often restricted by their potential adverse effects, particularly gastrointestinal, renal, and cardiovascular complications, which are of greater concern in older adults and in individuals with multimorbidity [4,5]. This scenario has stimulated growing interest in ‘natural’ anti-inflammatory molecules capable of modulating the inflammatory process with minimal or absent side effects.

**Figure 1:**
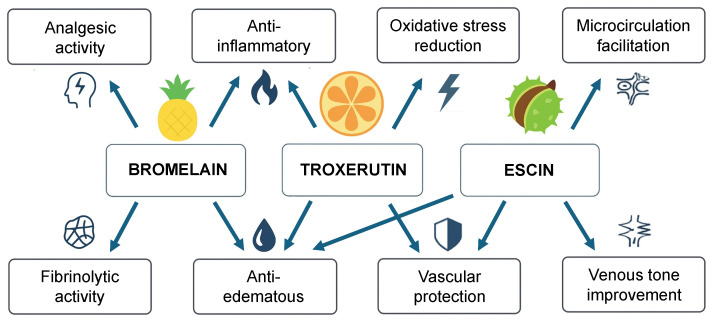
Overview of the main physiological actions of bromelain, troxerutin, and escin.

Among these, bromelain, a proteolytic enzyme complex extracted from the stem of *Ananas comosus* (pineapple), has shown promising results in both experimental and clinical settings. Bromelain exerts multiple biological effects, including anti-inflammatory, analgesic, anti-edematous, and fibrinolytic actions, primarily through modulation of bradykinin, prostaglandins, and cytokines [6,7,8,9]. Recent systematic reviews and clinical studies further support its ability to reduce postoperative pain and edema and to improve inflammatory markers [10,11]. Troxerutin, a semi-synthetic flavonoid derived from rutin, has demonstrated anti-edematous and vascular-protective properties, mainly by improving microvascular permeability and reducing oxidative stress [12]. By counteracting oxidative stress–related endothelial injury and sustaining microvascular function, its antioxidant activity may contribute to tissue recovery. These actions are consistent with mechanistic evidence showing inhibition of COX-2, NF-κB, and oxidative stress pathways [13]. Likewise, escin, a natural mixture of triterpenoid saponins obtained from *Aesculus hippocastanum* (horse chestnut), exhibits potent anti-inflammatory and anti-edematous effects by stabilizing capillary walls, reducing vascular leakage, and modulating leukocyte–endothelial interactions [14]. Its venotonic and endothelial-protective properties are also well documented by current evidence [15]. The main physiological activities of bromelain, troxerutin, and escin are summarized in Figure 1.

In the context of rehabilitation following major orthopedic surgery, these properties suggest that combined supplementation with bromelain, troxerutin, and escin may represent a valuable adjunct to conventional pharmacological approaches, supporting the reduction in post-operative pain and edema while facilitating faster recovery of physical function. Preliminary studies of bromelain supplementation conducted in surgical settings, such as dental and otorhinolaryngologic procedures, as well as in the management of sports-related injuries, have reported significant improvements in pain control and swelling [16,17]. Similarly, clinical studies evaluating troxerutin supplementation have documented reductions in vascular permeability, edema formation, and inflammatory markers in individuals with chronic venous insufficiency and after surgical procedures [13,18]. Escin has likewise been shown to reduce tissue edema and inflammation following orthopedic surgery and trauma-related interventions [19]. Despite this evidence, studies specifically examining the combined effects of these three compounds administered together after hip or knee arthroplasty remain limited.

In this observational study, we evaluated a commercially available formulation where these three agents are combined due to their complementary anti-inflammatory and anti-edematous actions. The aim of this study was to examine whether its use during early rehabilitation was associated with better pain control, reduced postoperative swelling, and improved functional recovery. This pilot work may therefore offer preliminary insights into the potential role of this formulation in supporting early postoperative recovery in this population.

## 2. Materials and Methods

### 2.1. Study Design

This was a retrospective observational study conducted in a post-acute rehabilitation unit and involving patients undergoing a standardized rehabilitation program after total hip or total knee arthroplasty. During routine clinical practice, the bromelain, troxerutin, and escin supplement was available for prescription at the discretion of the treating clinicians, so that patients could either receive it as part of usual care or follow standard management without supplementation. The primary aim of the study was to evaluate whether exposure to this combined supplement was associated with improved postoperative recovery, with specific focus on pain control, reduction in edema, and early functional gains during the rehabilitation phase. All participants provided written informed consent, and the study was conducted in accordance with the Declaration of Helsinki and was approved by the institutional ethics committee.

### 2.2. Participants

Eligible participants were adults aged 65 years or older admitted to the Geriatric Rehabilitation Unit of the Fondazione Policlinico Universitario “Agostino Gemelli” IRCCS (Rome, Italy) after elective total hip or total knee arthroplasty performed for severe and progressive osteoarthritis. Inclusion criteria comprised completion of primary prosthetic surgery without intra- or postoperative complications, hemodynamic stability, ability to participate in daily physiotherapy sessions, and absence of contraindications to bromelain-containing supplementation. Exclusion criteria included known allergy or hypersensitivity to pineapple or proteolytic enzymes, ongoing anticoagulant therapy (warfarin or direct oral anticoagulants), active peptic ulcer disease or gastrointestinal bleeding, uncontrolled hypertension, and severe hepatic or renal impairment.

### 2.3. Intervention

This study compared patients who received standard postoperative care with or without exposure to a bromelain-, troxerutin-, and escin-containing supplement. The control group received usual pharmacological management, including analgesics (paracetamol and, when required, NSAIDs), thromboprophylaxis, and a structured physiotherapy and occupational therapy program. Analgesic therapy followed routine postoperative practice. Paracetamol was generally used as first-line medication, while NSAIDs such as ketoprofen or ketorolac were added when clinically required. Doses and frequency were determined by the treating physicians according to individual patient needs. Patients in the supplemented group followed the same standard management and, in addition, were prescribed an oral formulation containing bromelain (400 mg/day), troxerutin (300 mg/day), and escin (40 mg/day), administered in two daily doses for up to 21 days. The supplement (BromeREX^®^, PharmaG, Anzio, Italy), standardized to provide 2500 GDU of bromelain activity, was administered within routine clinical practice under the supervision of healthcare professionals. The dosage reflected previous clinical evidence indicating anti-inflammatory and anti-edematous activity within this range [6,19]. Patients were classified retrospectively into the supplementation or control group based on whether the combined formulation had been prescribed as part of standard clinical practice. No randomization or predefined allocation criteria were used, and control patients were those managed with standard postoperative care during the same study period. Across both groups, postoperative analgesia followed the same standardized protocol used in the rehabilitation unit. Paracetamol represented the first-line medication and was administered in the majority of patients, typically at 1 g every 8 h (2–3 g/day), with treatment durations extending for approximately one to two weeks. NSAIDs were added when needed for insufficient pain control, generally for short periods and at low-to-moderate daily doses. The same analgesic protocol was applied across all patients, independent of group assignment.

### 2.4. Rehabilitation Program

Both groups followed the same multidisciplinary rehabilitation program, which began within 48 h after surgery. The protocol included assisted mobilization and gait training with gradual load progression, active and passive range-of-motion exercises, muscle strengthening, and proprioceptive and balance training. Each session lasted 60 min, twice daily, six days per week, and was conducted by the same team of physiotherapists and occupational therapists to ensure protocol uniformity and consistency across patients.

### 2.5. Outcome Measures

Patients were evaluated at admission to the rehabilitation unit (baseline, T0), after 10 days (T1), and after 21 days (T2). Pain intensity was assessed using the Visual Analog Scale (VAS, 0–10). Joint swelling was evaluated through circumferential measurements taken at standardized anatomical landmarks (10 cm above and below the patella for total knee arthroplasty, and mid-thigh and mid-leg for total hip arthroplasty). Range of motion (flexion and extension) was measured using a goniometer (GIMA, Milan, Italy). Functional performance was assessed with the Barthel Index and the six-minute walk test (seconds). At baseline, patients were evaluated shortly after transfer to the rehabilitation unit, when marked functional dependence was typically present following recent surgery. The Barthel Index was administered by experienced rehabilitation staff using a standardized protocol routinely adopted in the unit, ensuring procedural consistency. Adverse effects and treatment adherence were monitored throughout the study period.

### 2.6. Statistical Analysis

As this was a pilot study, the sample size was determined pragmatically based on feasibility considerations, with the aim of obtaining preliminary estimates to support the design of future studies. Characteristics of the study participants are presented according to exposure group. Descriptive statistics were computed for all variables. The normal distribution of continuous variables was assessed using the Kolmogorov–Smirnov test. Continuous variables are reported as mean ± SD, and categorical variables as absolute numbers (percentages). Differences between groups were evaluated using Fisher’s exact test for categorical variables and one-way analysis of variance (ANOVA) or the Kruskal–Wallis test for continuous variables, as appropriate. Time-series data collected at T0, T1, and T2 were analyzed according to the distributional characteristics of each variable. Continuous variables with approximately normal distribution were examined using repeated-measures ANOVA to assess within-patient changes over time, while variables with non-normal or ordinal distribution were evaluated using the Friedman test. Between-group comparisons at each time point followed the same approach described above. When ANOVA was followed by post hoc pairwise comparisons, Bonferroni-adjusted tests were applied; for non-parametric analyses, Dunn’s test was used. Statistical significance was set at *p* < 0.05. All analyses were performed using SPSS version 26.0 (IBM Corp., Armonk, NY, USA).

## 3. Results

### 3.1. Study Population

A total of 40 patients were enrolled and completed the study, 20 in the control group and 20 in the supplementation group (Table 1). The two groups were comparable at baseline in terms of age, sex distribution, type of surgery (hip versus knee arthroplasty), and preoperative functional measures. The mean age of the cohort was 69.4 ± 7.2 years, with a slight predominance of women (58%). No significant differences were observed between groups in any baseline characteristics.

### 3.2. Pain Reduction

**Figure 2:**
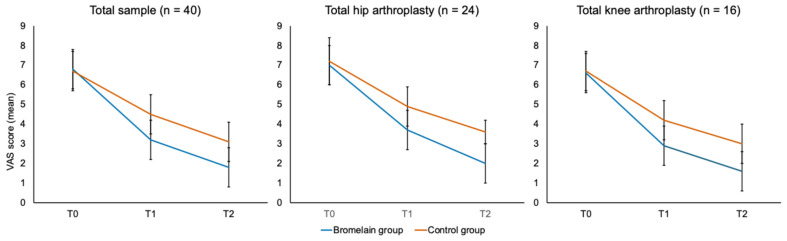
Pain intensity measured using the Visual Analog Scale (VAS) at baseline and follow-up (day 10 and day 21) in the supplementation and control groups.

Pain intensity progressively declined in both groups over the course of rehabilitation (Figure 2), with a markedly greater improvement among participants receiving the combined supplementation. At day 10 (T1), mean VAS scores decreased from 6.8 ± 1.0 to 3.2 ± 0.9 in the supplemented group, compared with 6.7 ± 1.1 to 4.5 ± 1.0 in controls (*p* < 0.01). By day 21 (T2), VAS scores further decreased to 1.8 ± 0.7 in the supplemented group and to 3.1 ± 0.8 in the control group (*p* < 0.001). These differences were consistent in both hip and knee arthroplasty subgroups. In patients undergoing THA, mean VAS scores declined from approximately 6.6 ± 1.1 at baseline to 3.6 ± 1.0 at day 10 and 2.2 ± 0.9 at day 21, with a steeper reduction among those receiving supplementation (6.6 ± 1.1 to 3.0 ± 0.9 and 1.7 ± 0.8) compared with controls (6.5 ± 1.1 to 4.2 ± 1.0 and 2.9 ± 0.8). A similar pattern was observed in TKA, where baseline pain was slightly higher, decreasing from about 7.1 ± 1.1 to 4.1 ± 1.0 at day 10 and 2.7 ± 0.9 at day 21; again, the supplemented group showed more pronounced improvement (7.2 ± 1.1 to 3.6 ± 0.9 and 2.0 ± 0.8) relative to controls (7.1 ± 1.1 to 4.9 ± 1.0 and 3.4 ± 0.9). In line with the greater reduction in pain, the duration of analgesic use was shorter among supplemented participants (11.9 ± 7.6 days) than in controls (15.9 ± 4.1 days).

### 3.3. Reduction in Edema

A summary of non-pain outcomes is presented in Table 2. Both groups showed a reduction in postoperative swelling, but the magnitude of improvement was significantly greater in the supplementation group. Among patients undergoing knee arthroplasty, the mean reduction in circumference 10 cm above the patella was 3.5 ± 0.6 cm with supplementation versus 2.1 ± 0.5 cm in the control group (*p* < 0.01). In hip arthroplasty patients, thigh circumference decreased by 2.9 ± 0.5 cm compared with 1.8 ± 0.4 cm in controls (*p* < 0.05).

### 3.4. Range of Motion

Range of motion improved over time in both groups, with significantly greater gains in those receiving supplementation. At day 21, knee flexion reached 100.2° ± 6.4° in the supplemented group versus 91.5° ± 7.2° in controls (*p* < 0.01). Similarly, hip flexion improved to 95.7° ± 5.9° in the supplementation group compared with 88.6° ± 6.1° in the control group (*p* < 0.05).

### 3.5. Functional Recovery

Functional outcomes showed patterns consistent with the improvements observed in pain, swelling, and mobility. The Barthel Index increased from 29.6 ± 9.0 at baseline to 58.2 ± 4.1 at day 21 in the supplemented group, and from 29.8 ± 7.9 to 50.4 ± 4.8 in the control group (*p* < 0.05). Performance on the six-minute walking test improved from 11.5 ± 23.6 s to 170.7 ± 72.7 s in the supplementation group, and from 11.8 ± 24.9 s to 150.4 ± 60.8 s in controls (*p* < 0.05).

### 3.6. Safety and Tolerability

No major or serious adverse events were reported. Two participants in the supplementation group experienced mild, transient gastrointestinal discomfort, which resolved without discontinuation. Adherence to rehabilitation and medical treatments exceeded 95% in both groups.

## 4. Discussion

The present study indicates that oral supplementation with bromelain, troxerutin, and escin may support postoperative recovery in older adults undergoing total hip or total knee arthroplasty. Compared with standard care alone, patients exposed to the combined formulation showed earlier pain reduction, a more marked decrease in edema, and improved functional performance during early rehabilitation. Although preliminary, these observations are consistent with the pharmacological properties of the three components, which exert complementary actions that may contribute to postoperative recovery.

Bromelain has well-described analgesic and anti-inflammatory effects mediated through modulation of the kallikrein–kinin and prostaglandin systems, resulting in reduced bradykinin formation and attenuation of local inflammatory responses [20,21,22]. It also downregulates pro-inflammatory cytokines such as TNF-α, IL-1β, and IL-6 while promoting anti-inflammatory mediators [6]. At the microvascular level, bromelain may enhance lymphatic drainage and tissue oxygenation, facilitating edema reabsorption [23]. Its fibrinolytic and antithrombotic activities are relevant in the postoperative orthopedic setting, where micro-thrombotic changes may contribute to persistent swelling [24,25].

Troxerutin, a flavonoid derived from rutin, exhibits antioxidant, anti-inflammatory, vasculoprotective, and metabolic actions [13,26]. Following partial biotransformation by gut microbiota, it enhances endogenous antioxidant systems, including glutathione and key enzymes such as superoxide dismutase, catalase, and glutathione peroxidase [13]. Through the mitigation of oxidative stress and preservation of cellular integrity, troxerutin may help support tissue recovery in the postoperative phase [13]. Escin, a triterpenic saponin extracted from *Aesculus hippocastanum*, stabilizes capillary walls, reduces vascular permeability, and modulates inflammatory mediators, thereby improving microcirculatory flow and promoting tissue fluid clearance [15]. These effects are particularly relevant after arthroplasty, where postoperative edema and microvascular dysfunction can hinder mobility and delay rehabilitation progress. Taken together, the improvements in pain, swelling, and functional performance observed in the supplemented group suggest that this formulation could serve as an adjunctive measure during early rehabilitation. These findings are consistent with previous reports documenting beneficial effects of bromelain, troxerutin, and escin when used individually in trauma, postoperative inflammation, and soft-tissue injuries [27], and extend such observations to older rehabilitation patients. The favorable tolerability profile observed in the present cohort is also noteworthy given the frequency of multimorbidity and polypharmacy in this population. Although the three agents have complementary mechanisms that may plausibly result in additive or synergistic effects, direct clinical evidence confirming pharmacodynamic synergy remains limited. A randomized postoperative study evaluating a fixed-dose combination of bromelain, escin, and a flavonoid derivative reported greater improvements in pain and inflammatory markers compared with placebo and an active comparator [28], although single-agent arms were not included. Similar findings have been observed in prospective studies of multi-ingredient formulations containing bromelain and escin [29], and in other enzyme-based trials [30]. Overall, current evidence supports the complementary actions of these agents, while the question of true synergy remains to be specifically addressed in future studies.

A number of considerations should be taken into account when interpreting these findings. First, the sample size was modest and consistent with a pilot design, and the single-center setting may limit generalizability. Second, the observational nature of the study does not allow causal inference, and pain assessment may have been influenced by the absence of blinding. Third, allocation to supplementation or standard care reflected routine clinical practice rather than randomization; despite comparable baseline characteristics, residual selection bias cannot be fully excluded. Fourth, postoperative analgesic regimens were individualized according to clinical needs, preventing standardized comparisons of medication use between groups. Fifth, although descriptive pain trajectories were examined separately for hip and knee arthroplasty, the sample was insufficient to support adequately powered inferential comparisons or stratified multivariable analyses. Sixth, because the study relied on routinely collected clinical data, mechanistic biomarkers such as TNF-α, IL-1β, and IL-6 were not available, limiting insight into potential biological pathways influenced by supplementation. Finally, the study was not designed to assess pharmacodynamic synergy among the three agents. Larger randomized multicenter trials, including standardized analgesic protocols and mechanistic evaluations, are required to confirm these preliminary observations and clarify optimal dosing strategies and long-term outcomes.

Despite these limitations, the natural origin and pleiotropic biological actions of bromelain, troxerutin, and escin make them of potential interest as supportive agents in postoperative rehabilitation. Their combined anti-inflammatory, anti-edematous, and fibrinolytic activities offer a plausible physiological rationale for their use in this context [29,31,32,33]. From a practical standpoint, these preliminary findings suggest that bromelain-, troxerutin-, and escin-based supplementation may represent a simple and well-tolerated adjunctive option to support postoperative recovery during early rehabilitation, particularly in older adults for whom NSAIDs use may be limited. Within real-world rehabilitation settings, such an approach could help reduce pain, attenuate edema, and facilitate functional gains without increasing the pharmacological burden. However, these implications should be viewed as hypothesis-generating and require confirmation in larger, controlled studies to better define the role of such supplementation within standardized postoperative rehabilitation pathways.

## 5. Conclusions

In this pilot observational study, supplementation with bromelain, troxerutin, and escin was associated with reduced postoperative pain and edema, and with improvements in functional measures during early rehabilitation after hip or knee arthroplasty in older adults. These observations, derived from routine clinical practice, provide preliminary evidence that should be interpreted within the constraints of the observational study design. Controlled randomized trials will be necessary to validate these findings, better define their generalizability, and clarify optimal timing, duration, and patient selection.

## Figures and Tables

**Table 1:** Characteristics of the study population according to intervention group.

Characteristics	Total Sample (*n* = 40)	Bromelain Group (*n* = 20)	Control Group (*n* = 20)
Age, years	69.3 ± 7.2	67.9 ± 7.1	70.7 ± 7.4
Sex, female	23 (58%)	12 (60%)	11 (55%)
Days from surgery to RU admission	7.5 ± 3.3	8.1 ± 2.7	6.9 ± 3.2
Type of surgery			
Total hip arthroplasty	24 (60%)	13 (65%)	11 (55%)
Total knee arthroplasty	16 (40%)	7 (35%)	9 (45%)
Number of diseases	4.7 ± 3.0	4.9 ± 3.2	4.5 ± 2.9
Number of medications	3.3 ± 2.6	3.7 ± 3.0	2.9 ± 2.3
BMI, kg/m^2^	26.9 ± 3.9	26.6 ± 3.9	27.3 ± 3.3
Pain			
VAS at rest position	2.9 ± 2.2	2.8 ± 2.4	3.1 ± 2.7
VAS after exercise	5.0 ± 2.2	4.8 ± 2.5	5.3 ± 1.9
Barthel index	29.7 ± 8.9	29.6 ± 9.0	29.8 ± 7.9
6 min walk test, s	11.6 ± 24.1	11.5 ± 23.6	11.8 ± 24.9

Data are reported as number (percentage) for sex and type of surgery. All other variables are presented as mean ± SD. BMI, body mass index; RU, rehabilitation unit; VAS, Visual Analog Scale.

**Table 2:** Summary of changes (delta values) in non-pain outcomes between baseline and day 21 in the supplementation and control groups.

Outcome	Bromelain Group (*n* = 20)	Control Group (*n* = 20)	*p*
Knee circumference (10 cm above patella), cm	–3.5 ± 0.6	2.1 ± 0.5	<0.01
Thigh circumference (hip arthroplasty), cm	–2.9 ± 0.5	–1.8 ± 0.4	<0.05
Knee flexion, degrees	+100.2 ± 6.4	+91.5 ± 7.2	<0.01
Hip flexion, degrees	+95.7 ± 5.9	+88.6 ± 6.1	<0.01
Barthel Index	+28.6 ± 9.9	+20.6 ± 9.2	<0.05
Six-minute walking test	+159.2 ± 76.4	+138.6 ± 65.7	<0.05

Values represent changes (delta) between baseline and day 21. Range of motion (knee flexion and hip flexion) outcomes are reported as day-21 values.

## Data Availability

The data that support the findings of this study are available from the corresponding author upon reasonable request.
